# MicroRNA-937 is overexpressed and predicts poor prognosis in patients with colon cancer

**DOI:** 10.1186/s13000-019-0920-3

**Published:** 2019-12-19

**Authors:** Huiya Liu, Lin Ma, Ling Wang, Yizuo Yang

**Affiliations:** 1 Department of Gastroenterology, Heze Municipal Hospital, Heze, 274400 Shandong China; 2 Department of Laboratory Medicine, Heze Municipal Hospital, Heze, 274400 Shandong China; 3 Department of Cardiac Intervention, Heze Municipal Hospital, Heze, 274400 Shandong China; 4 Department of Geriatrics, Heze Municipal Hospital, No. 2888, Caozhou Road, Heze, 274400 Shandong China

**Keywords:** miR-937, Colon cancer, Prognosis, Proliferation, Migration, Invasion

## Abstract

**Background:**

Colon cancer is a heterogeneous tumor and a leading cause of cancer-related mortality. MicroRNA (miRNA) has been proposed as the biomarker in cancers. The aim of this study was to investigate the clinical significance and potential functional role of miR-937 in colon cancer.

**Methods:**

In the present study, reverse transcription-quantitative polymerase chain reaction (qRT-PCR) was conducted to examine the expression levels of miR-937 in colon cancer tissues and cell lines. Kaplan-Meier curve and Cox regression analyses were used to determine the prognostic impact of miR-937 on survival. Cell Counting Kit-8 and Transwell assays were performed to examine cell proliferation, migration, and invasion, respectively.

**Results:**

miR-937 was significantly upregulated in colon cancer tissues and cell lines. Clinical analysis results showed that miR-937 expression was associated with lymph node metastasis and TNM stage. Patients with high miR-937 expression predicted a shorter overall survival rate. Functionally, overexpression of miR-937 promoted cell proliferation, migration, and invasion, while inhibition of miR-937 inhibited these cellular behaviors in vitro.

**Conclusions:**

These results suggested that miR-937 may act as a prognostic biomarker and a potential target for therapeutic strategy, as well as promote proliferation, migration, and invasion of colon cancer.

## Introduction

Colon cancer is one of the most common malignant tumors of the digestive tract and the leading cause of cancer-related mortality worldwide [1, 2]. The development of colon cancer is influenced by many factors and is a multi-gene related, multi-step complex process [3]. Colon cancer patients at early stages often have long-term survival, however, colon cancer with early-onset symptoms is not typical and the patients usually initially diagnosed at advanced stages with unsatisfactory prognosis [4]. Despite considerable progress in current therapeutic strategies, such as surgery, chemoradiotherapy, and comprehensive biological treatment, the overall survival rate of advanced colon cancer is still unsatisfactory [5]. Therefore, better cancer-related biomarkers and therapeutic targets for the treatment of colon cancer are necessary.

Numerous studies have demonstrated that abnormal expression of microRNAs (miRNAs) is closely linked to the initiation and development of cancers [6]. MiRNA is a family of endogenous small non-coding RNAs of 19–24 nucleotides in length and negatively regulate gene expression at the post-transcriptional level by binding to the 3′-UTR of their target mRNA [7, 8]. Recently, increasing evidence indicated that miRNAs are involved in various biological behaviors, including cell proliferation, differentiation, migration, invasion, and apoptosis [9, 10]. And miRNAs, including miR-378 [11], miR-590-3p [12], and miR-34b [13], are abnormally expressed and function as oncogenic or suppressor role in colon cancer. A previous study of miRNA expression profile showed miR-937 is one of the upregulated miRNAs in colorectal cancer [14]. However, the potential role of miR-937 has not yet completely understood in colon cancer.

The present study attempt to detect whether the miR-937 expression is upregulated in colon cancer and examine its clinical significance. The functional roles of miR-937 in the progression of colon cancer were also explored.

## Materials and methods

### Patients and tissue specimens

The present study was approved by the Ethics Committee of Heze Municipal Hospital. All of the 109 patients with colon cancer involved in this study provided written informed consent prior to sample collection. Among them, 109 paired fresh tumor tissue specimens and adjacent non-tumor tissue specimens were collected during surgical resection at Heze Municipal Hospital between June 2010 and December 2013. All tissue specimens were confirmed by experiment pathologists and immediately put into liquid nitrogen until RNA extraction after surgery. All patients who provided tissue specimens had not been treated with preoperative radiotherapy and chemotherapy. The corresponding patient characteristics were collected and listed in Table 1. The 5-year survival information was also collected and recorded for subsequent analysis.

**Table 1:** Association of clinical characteristics of colon cancer patients and miR-937 expression

Characteristics	Cases No. (*n* = 109)	miR-937 expression		*P*
		Low (*n* = 52)	High (*n* = 57)	
Age				0.510
≤ 60	56	25	31	
> 60	53	27	26	
Gender				0.726
Female	38	19	19	
Male	71	33	38	
Differentiation				0.348
Well - Moderate	62	32	30	
Poor	47	20	27	
Lymph node metastasis				0.026
Negative	57	33	24	
Positive	52	19	33	
Vascular invasion				
Negative	60	33	27	0.092
Positive	49	19	30	
Histological type				
Adenocarcinoma	86	42	44	0.648
Mucinous/signet-ring cancer	23	10	13	
TNM stage				0.016
I - II	56	33	23	
III - IV	53	19	34	

### Cell culture and transfection

Colon cancer cell lines (LoVo, SW620, CW-2, HCT116) and the normal colonic epithelial cell line NCM460 were purchased from the Shanghai Institute of Biochemistry and Cell Biology (Shanghai, China). All cells were cultured in DMEM (Gibco, Grand Island, NY, USA) with 10% FBS (Invitrogen, CA, USA) in a humidified atmosphere at 37 °C with 5% CO_2_.

The miR-937 mimics, inhibitors, and their negative controls (NCs; mimic NC and inhibitor NC) were purchased from Shanghai GenePharma Co. Ltd. (Shanghai, China). The transfection procedure was performed using Lipofectamine 3000 (Invitrogen; Thermo Fisher Scientific, USA) following the manufacturer’s instructions. After transfection 48 h, reverse transcription-quantitative polymerase chain reaction (qRT-PCR) was used to detect the transfection efficiency.

### RNA extraction and qRT-PCR

RNA was extracted from tissue specimens and cultured cells with TRIzol reagent (Invitrogen; Thermo Fisher Scientific, Inc.). Complementary DNA (cDNA) was synthesized using a TaqMan MicroRNA Reverse Transcription kit (Applied Biosystems; Thermo Fisher Scientific, Inc.). Subsequently, qRT-PCR was performed to determine miR-937 expression using SYBR Green Mix (Roche Diagnostics, Mannheim, Germany) using a 7300 real-time PCR system (ABI). The relative gene expression was calculated using the 2^-ΔΔCt^ method and normalized to U6.

### Cell proliferation assay

A density of 2000 cells/well of transfected cells was seeded into 96 well plates, and cell proliferation was measured using Cell Counting Kit-8 (CCK-8, Dojindo, Japan) according to the manufacturer’s protocol. In brief, 10 μl CCK-8 solution was added to each well at 0, 24, 48, and 72 h after seeding, and the solution was incubated for 2 h. The absorbance in each well was measured at 450 nm using a microplate reader (Bio-Rad Laboratories, Inc., Hercules, CA, USA).

### Cell migration and invasion assays

Cell migration and invasion assays were conducted using a Transwell chamber (8 μm pores; BD Biosciences, Franklin Lakes, NJ, USA). 5 × 10^4^ transfected cells in FBS-free medium were placed into the upper chambers of 24-well plates with or without Matrigel (BD Biosciences, Franklin Lakes, NJ, USA), and the lower chambers contained culture medium with 10% FBS. Matrigel was only used for cell invasion assays. The cells were incubated at 37 °C for 24 h, then, the migrated and invaded cells were fixed and stained. Finally, the stained cells from 5 random fields were counted under a light microscope.

### Statistical analysis

All experiments were performed at least three times, and data were presented as the mean ± SD. SPSS 20.0 statistical software (SPSS, Chicago, IL, USA) and GraphPad 5.0 (GraphPad Software, La Jolla, CA, USA) were used to process statistical analyses. The χ^2^ test, Student’s t-test, and one-way ANOVA were used to make comparisons. Kaplan-Meier analysis and multivariate survival analysis were performed to analyze the prognostic significance of miR-937 in colon cancer. *P* < 0.05 was considered to be a statistically significant difference.

## Results

### miR-937 expression levels in colon cancer patients and cell lines

**Fig. 1:**
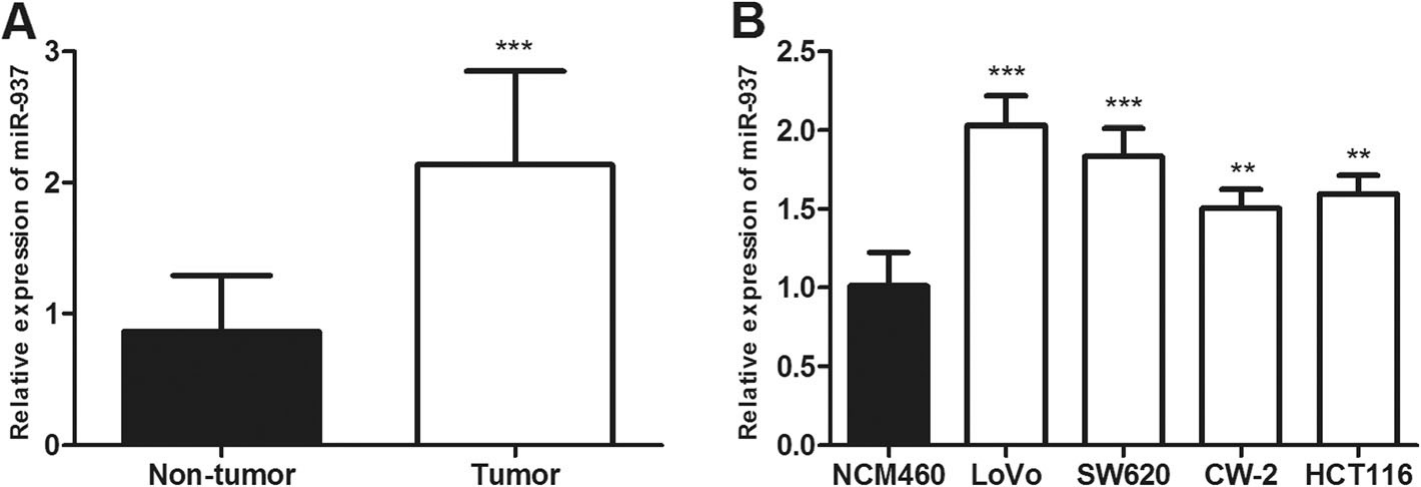
The miR-937 expression is detected in colon cancer tissues and cell lines via qRT-PCR analysis. **a**. miR-937 expression is increased in colon cancer tissues compared with adjacent normal tissues. **b**. miR-937 expression is upregulated in colon cancer cells (LoVo, SW620, CW-2, HCT116) compared with a normal colonic epithelial cell line (NCM460). **P < 0.01, ***P < 0.001

The expression levels of miR-937 in tumor and adjacent normal tissue specimens of 109 colon cancer patients were detected by the qRT-PCR assay. The results showed that the relative expression of miR-937 in tumor tissues was significantly higher than in the adjacent normal tissues (*P* < 0.001, Fig. 1a).

Then, the expression levels of miR-937 were determined in colon cancer cell lines (LoVo, SW620, CW-2, HCT116) and a normal colonic epithelial cell line NCM460. Among colon cancer cell lines, LoVo and SW620 had a relatively higher miR-937 expression than the other two colon cancer cell lines CW-2 and HCT116. Therefore, both LoVo and SW620 cells were selected to assess the effects of miR-937 overexpression or silencing. In all, miR-937 was revealed to be significantly upregulated in the colon cancer cells compared with that in the normal colonic epithelial cells NCM460 (*P* < 0.001, Fig. 1b).

### Relationship between the expression levels of miR-937 and the clinicopathological characteristics of patients

To investigate the clinical role of miR-937 in colon cancer, all enrolled colon cancer patients were divided into miR-937 low or high expression groups based on the mean value of miR-937. Overexpression of miR-937 was illustrated to be significantly associated with lymph node metastasis (*P* = 0.026) and TNM stages (*P* = 0.016) of patients with colon cancer (Table 1). However, the relative expression of miR-937 in tumor tissues of colon cancer patients does not correlate with age, gender, differentiation, vascular invasion, and histological type (*P* > 0.05).

### High expression of miR-937 is associated with a poor survival rate of patients with colon cancer

**Fig. 2:**
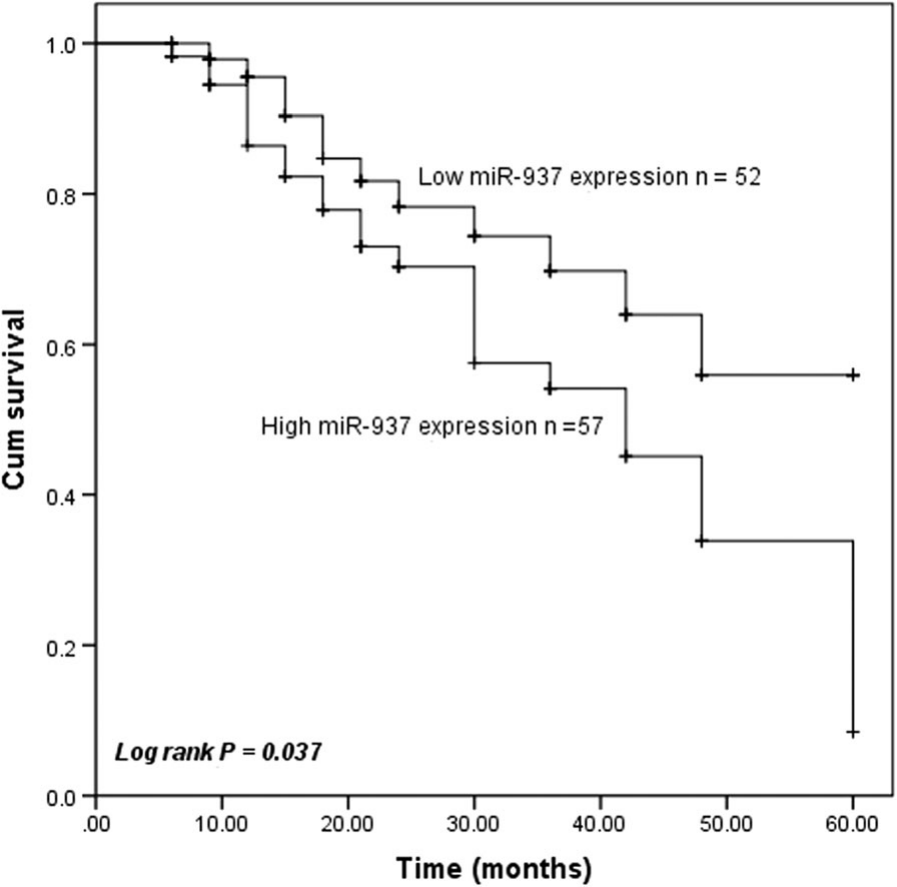
Kaplan-Meier analysis showed that colon cancer patients with high miR-937 expression had shorter overall survival rate. The log-rank test was applied, *P* = 0.037

To further explore the prognostic significance of miR-937 in colon cancer, all patients enrolled were followed up for 5 years, the survival rate was recorded. According to the survival information, the Kaplan-Meier curve was plotted. The results showed that the 5-year survival rate of patients in the low miR-937 expression group was obviously higher than that of patients in the high miR-937 expression group (log-rank *P* = 0.037, Fig. 2). Then multivariate Cox’s proportional hazard model analysis results identified that lymph node metastasis (HR = 2.190, 95%CI: 1.056–4.540, *P* = 0.035), vascular invasion (HR = 2.739, 95%CI: 1.089–6.886, *P* = 0.032), TNM stage (HR = 2.364, 95%CI: 1.055–5.298, *P* = 0.037) and miR-937 expression (HR = 2.449, 95%CI: 1.195–5.020, *P* = 0.014) were independent risk factors for survival (Table 2). Collectively, the data suggested that miR-937 may be a potential prognostic biomarker for the patient with colon cancer.

**Table 2:** Multivariate Cox analysis for overall survival of colon cancer patients

Characteristics	Multivariate analysis		
	HR	95% CI	*P*
miR-937	2.449	1.195–5.020	0.014
Age	0.607	0.304–1.213	0.157
Gender	1.741	0.857–3.536	0.157
Differentiation	0.561	0.275–1.146	0.113
Lymph node metastasis	2.190	1.056–4.540	0.035
Vascular invasion	2.739	1.089–6.886	0.032
Histological type	0.609	0.268–1.384	0.236
TNM stage	2.364	1.055–5.298	0.037

### Overexpression of miR-937 enhanced the proliferation, migration, and invasion of colon cancer cells in vitro

**Fig. 3:**
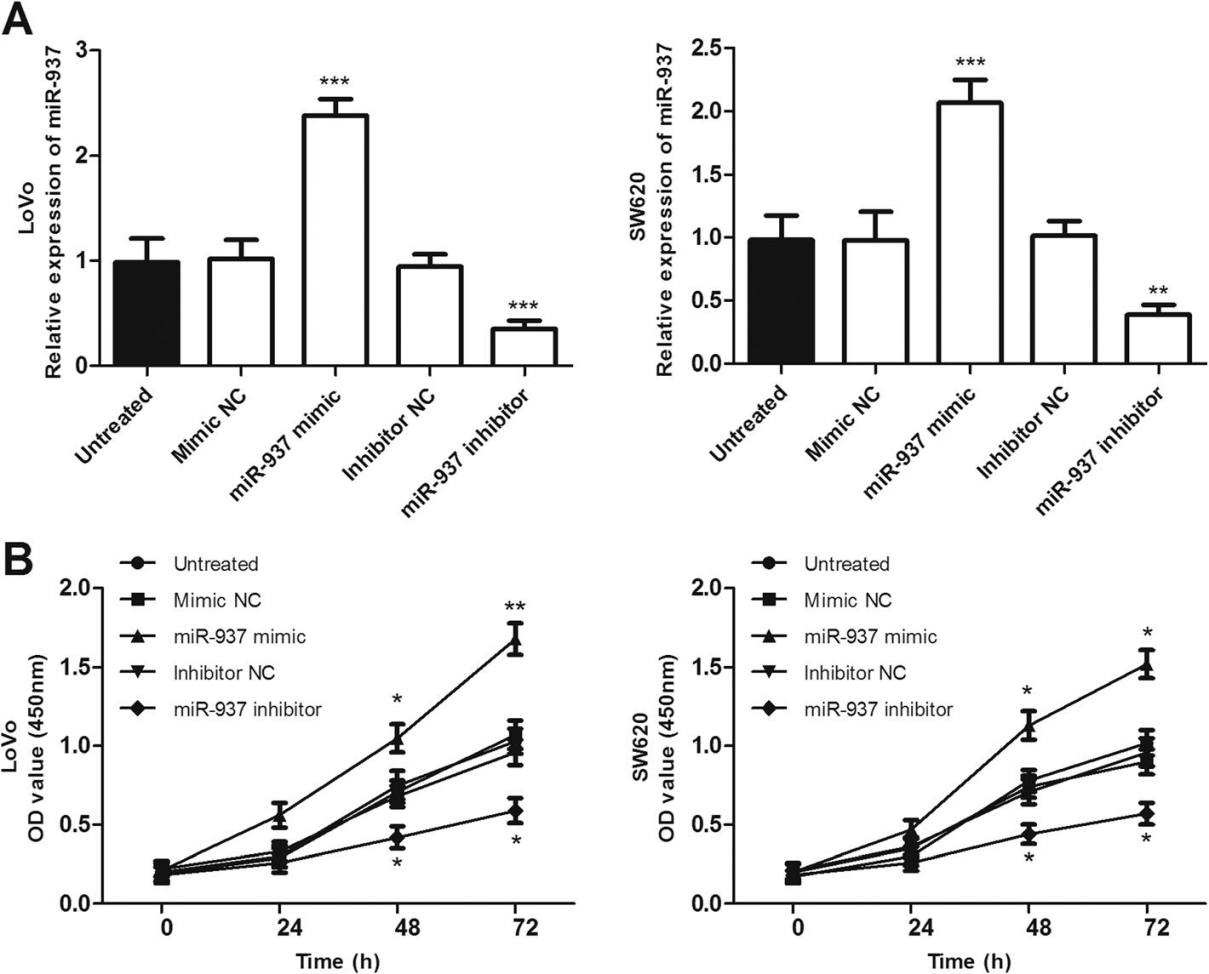
Overexpression and downregulation of miR-937 promotes and inhibits the proliferation of colon cancer cells (LoVo and SW620), respectively, compared with untreated cells. **a**. The relative expression level of miR-937 was significantly increased by miR-937 mimic, while decreased by miR-937 inhibitor in LoVo and SW620 cells, compared with that in the untreated cell. **b**. Cell proliferation was detected by CCK-8 assay. *P < 0.05, **P < 0.01, ***P < 0.001

As miR-937 expression was observed upregulated in colon cancer tissues and cells, it was hypothesized that miR-937 might act as an oncogenic role in colon cancer. To investigate the biological function of miR-937 on the proliferation, migration, and invasion of colon cancer cells in vitro, we constructed miR-937 overexpression or knockdown colon cancer cell lines using miR-937 mimics or inhibitors in LoVo and SW620 cells. The qRT-PCR confirmed the transfection efficiency (*P* < 0.001, Fig. 3a). CCK-8 assay results showed that miR-937 overexpression notably enhanced cell proliferation, while inhibition of miR-937 suppressed cell proliferation compared with untreated control cells (*P* < 0.05, Fig. 3b).

**Fig. 4:**
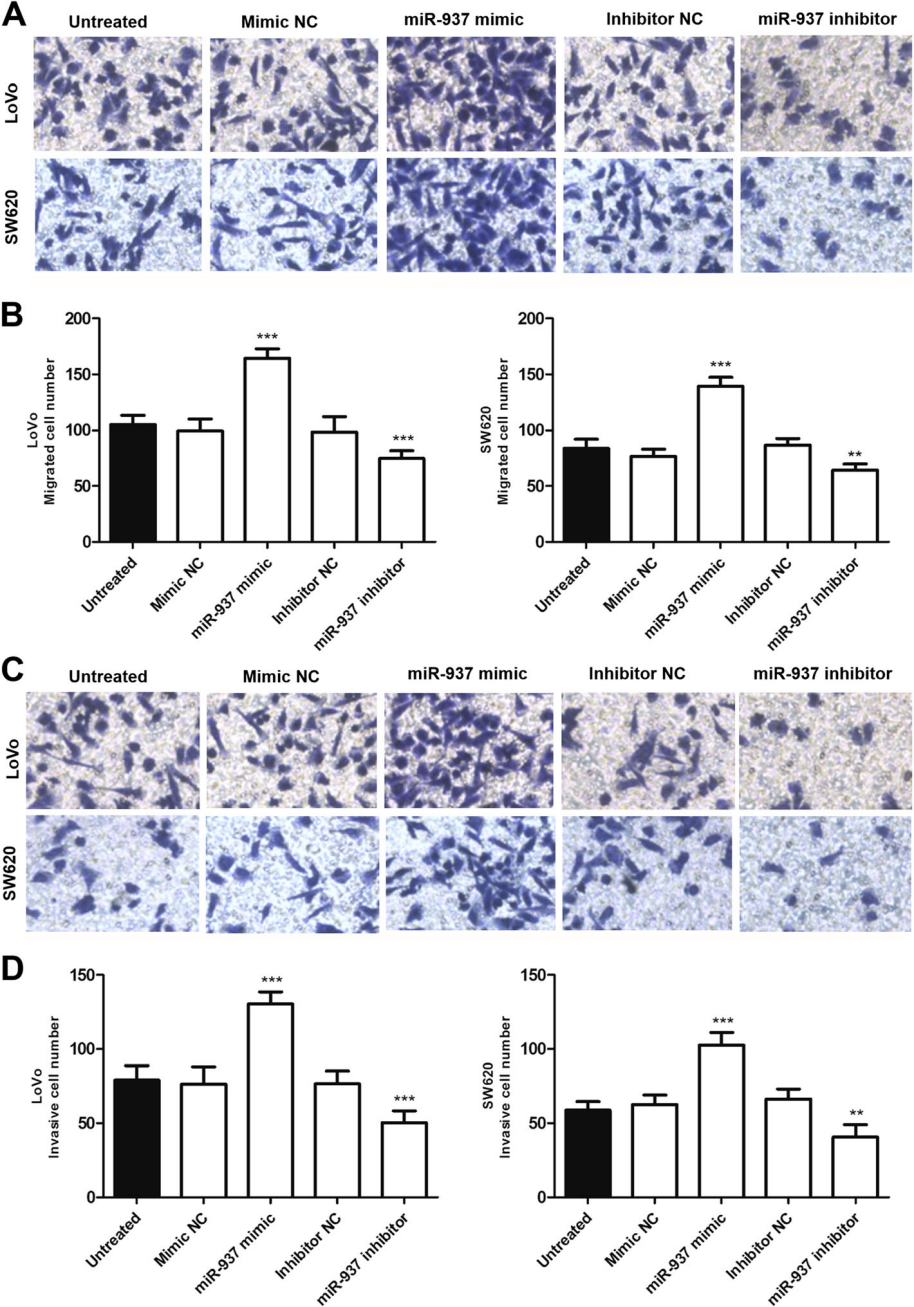
Overexpression of miR-937 promotes the migratory and invasive capabilities, while the downregulation of miR-937 inhibits the migratory and invasive capabilities of colon cancer cells (LoVo and SW620), compared with untreated cells. **a**. Representative images of Transwell migration assay (magnification × 200). **b**. Transwell migration assay was applied to investigate migration ability. **c**. Representative images of Transwell invasion assay (magnification × 200). **d**. Transwell invasion assay was applied to investigate invasion ability. **P < 0.01, ***P < 0.001

Results of Transwell assay with or without Matrigel showed that overexpression of miR-937 promoted cell migration and invasion, while knockdown of miR-937 inhibited cell migration and invasion compared with untreated cells (*P* < 0.01, Fig. 4a–d).

## Discussion

In recent years, more and more studies demonstrated that miRNAs play a vital role in the regulation of cancer progression, including colon cancer [15–17]. For instance, aberrant expression of miRNAs, such as miR-141-3p [17], miR-193b [18], and miR-613 [19], acts as an oncogene or tumor suppressor in regulating colon cancer cell proliferation, migration, and invasion. Notably, miRNAs have been suggested as diagnostic and/or prognostic biomarkers, as well as effective therapeutic targets for the treatment of cancer [20–22]. Several miRNAs have been identified to be independent prognostic factors for patients with colon cancer, such as miR-181-3p [23] and miR-873-5p [24]. Hence, identification of colon cancer-specific miRNAs are crucial for understanding their role in colon cancer progression and may be crucial for defining novel therapeutic targets.

In the present study, our results showed that the expression level of miR-937 was significantly upregulated in colon cancer tissue specimens. The upregulation of miR-937 was remarkably associated with lymph node metastasis and TNM stage. And colon cancer patients with high miR-937 expression had a shorter overall survival rate. In addition, the expression levels of miR-937 were also higher in colon cancer cell lines. Upregulation or downregulation of miR-937 promoted or inhibited the proliferation, migration, and invasion capacities of colon cancer cells in vitro.

Consistent with the results in this study, miR-937 was identified upregulated in lung cancer [25], and multiple myeloma [26]. On the contrary, miR-937 was downregulated in gastric cancer [27]. These inconsistent observations revealed the expression pattern of miRNA in cancers may depend on cancer types. Herein, we measured miR-937 expression in colon cancer tissues and cell lines using qRT-PCR assay and the results revealed that miR-937 expression was high in colon cancer tissues. The high miR-937 expression was significantly associated with lymph node metastasis and TNM stage of colon cancer patients. In addition, miR-937 was also upregulated in colon cancer cells. These results suggest that the upregulation of miR-937 may be a frequent event in the development of colon cancer. In addition, colon cancer patients with high miR-937 expression showed shorter overall survival than those patients with low miR-937 expression. Multivariate Cox regression analysis results also showed lymph node metastasis, vascular invasion, and TNM stage were independent prognostic factors in colon cancer, which is consistent with previous studies [28, 29]. Importantly, multivariate Cox regression analysis results showed miR-937 was an independent prognostic factor in colon cancer. These results suggested that miR-937 may be a prognostic biomarker of colon cancer, which functioned similarly to several miRNAs, such as miR-613 [19] and miR-185 [30]. For instance, miR-185 may serve as a prognostic factor and inhibits migration and invasion by targeting Wnt1 in colon cancer [30].

The aberrant expression of miRNAs has been identified to be involved in the development and progression of cancers, including colon cancer [31]. Yu and colleagues found that miR-937 was downregulated and inhibited cell proliferation and metastasis in gastric cancer by targeting FOXL2, which might be a potential target for the treatment of gastric cancer [27]. In this study, we also investigated the potential functional role of miR-937 in colon cancer cells in vitro. The results showed that overexpression of miR-937 promoted proliferation, migration, and invasion of colon cancer cells, which suggested miR-937 might play an oncogenic role in colon cancer. In lung cancer, miR-937 was upregulated and contributed to cell proliferation by inhibiting INPP4B, it might be a valuable target for lung cancer therapy [25]. In breast cancer, Fang et al. showed miR-937 was upregulated and regulated the proliferation and apoptosis through targeting APAF1 [32]. In the present study, miR-937 was identified upregulated in colon cancer and promoted proliferation, migration, and invasion of colon cancer cells. In future experiments, the potential target genes and the detailed molecular mechanism will be explored in colon cancer. Additionally, there are still some limitations in our study. Firstly, the sample size is limited. Moreover, the clinical characteristics information included in the present study was not comprehensive. For example, tumor budding information was not taken into account in these colon cancer patients. In the future study, more clinical information will be included and a larger study population will be required to confirm the present results.

Taken together, the current study demonstrated that miR-937 was upregulated in colon cancer tissues and cells, as well as was significantly associated with several clinical features and overall survival rate of colon cancer patients. The miR-937 enhanced the proliferation, migration, and invasion of colon cancer cells. These results suggested that miR-937 may be a prognostic biomarker and a potential therapeutic target for the treatment of colon cancer.

## Data Availability

All data generated or analyzed during this study are included in this published article.

## Abbreviations

CCK-8: Cell Counting Kit-8
cDNA: complementary DNA
miRNA: microRNA
miRNAs: microRNAs
NCs: Negative controls
qRT-PCR: reverse transcription-quantitative polymerase chain reaction
